# “It’s a precious gift, not to waste”: is routine cross matching necessary in orthopedics surgery? Retrospective study of 699 patients in 9 different procedures

**DOI:** 10.1186/s12913-018-3613-9

**Published:** 2018-10-20

**Authors:** Obada Hasan, Eraj Khurshid Khan, Moiz Ali, Sadaf Sheikh, Anam Fatima, Haroon U. Rashid

**Affiliations:** 1 Department of surgery, Section of Orthopaedics, The Aga Khan University Hospital (AKUH), National stadium road, P.O. Box 3500, Karachi, 74800 Pakistan; 2 The Aga Khan University Hospital (AKUH), Karachi, Pakistan; 3 Emergency medicine, The Aga Khan University Hospital (AKUH), Karachi, Pakistan; 4 Neurosurgery Department, Karachi, Pakistan

**Keywords:** Blood crossmatch, Blood transfusion, CT ratio, MSBOS, Orthopedics

## Abstract

**Background:**

Orthopedic surgeries are usually associated with excessive blood loss which leads surgeons to overestimate need for blood transfusions and over ordering of blood. The cross matched blood, when not used, leads to the wastage of blood bank resources in terms of time, money and manpower. The objective of this study was to investigate the compliance to previously proposed MSBOS and to provide updated recommendations for all orthopedic procedures.

**Methods:**

A retrospective analysis was conducted between 1st June 2015 and 31st May 2016. Patients admitted to the orthopedic surgery service for whom blood products were requested were included. Cross Match/Transfusion (CT) Ratio, Transfusion Index and Transfusion Probability were calculated. Values of < 2.5, > 0.5 and > 30% respectively, were taken as standards. Maximum Surgical Blood Ordering Schedule (MSBOS) was proposed based upon these calculations using Mead’s criteria.

**Results:**

Six hundred and ninety-nine patients were sampled after implementing exclusion criteria. The overall CT ratio was 4.87, transfusion index was 0.55 and transfusion probability was 25%. A compliance rate of 24.6% was observed with the reference CT ratio of 2.5. Highest CT ratio was calculated for arthroscopic procedures while tumor resection had the lowest ratio. Age, procedure performed, ASA status and use of tourniquet were found to be significantly associated with CT ratio being greater or less than 2.5.

**Conclusion:**

Results showed significant wastage of blood products and non-compliance with blood ordering guidelines. Hence there is need for large scale prospective studies to establish MSBOS and ensure its compliance.

## Background

Orthopedic procedures are usually associated with excessive amounts of blood loss and subsequent blood transfusions, hence leading surgeons to overestimate the need of blood transfusion during and after surgery [4, 21]. The cross matched blood, when not used, leads to the wastage of blood bank resources in terms of time, money and manpower. Furthermore, blood also becomes unavailable to other deserving patients [4, 14].

The increased demands of blood products combined with increasing costs has led to conduction of large scale surveys and evaluation of blood requests and transfusion practices. Subsequently, the studies have shown considerable over ordering of blood products and wastage of this precious commodity [8, 12, 14]. As blood transfusions play a vital role in resuscitation of surgical patients, there exists a need to develop specific criterion for blood ordering practices in elective surgeries, so that it is easily available for all patients when needed [8].

American Society of Anesthesiologists (ASA) has recommended the use of a maximum surgical blood order schedule (MSBOS) in accordance with institutional policy, as a strategy to improve the efficiency of blood ordering practices [6, 10]. At our institution. The Aga Khan University Hospital (AKUH), in 2001, Chawla et al. analyzed the practice of ordering blood for various elective procedures and they recommended that MSBOS should be revised yearly to keep it responsive to the changing needs [5].

Our aim was to investigate the compliance to previously established MSBOS and to provide recommendations for all orthopedic procedures, including the ones not mentioned in the previous study.

## Methods

A retrospective analysis was conducted for the period between 1st June 2015 and 31st May 2016. The study included all patients who underwent an orthopedic procedure and for whom 1 or more units of packed cell volume (PCV) were cross matched pre-operatively. As per our institutional policies, the operating surgeon was responsible for ordering of blood products pre-operatively. Patients who underwent day care procedures, who had a pre-operative hemoglobin (Hb) level of < 9 g/dl or who had known coagulation disturbances or were on blood thinners were excluded. In addition, patients who underwent massive blood transfusion i.e. > 10 units of PCV were also excluded to eliminate biased results [13]. With regards to specific procedures, revision arthroplasty cases were excluded. While for tumor resections, only malignant tumors (Enneking’s stage IB and IIB) which were treated with wide margin excision were included. All patient information was recorded, including: demographics, ASA level, procedure performed, application of tourniquet, nature of surgery i.e. emergency or elective and units of PCV cross-matched and transfused.

For the purpose of this study, wound debridement procedures were divided into 2 categories; major and minor. Major wound debridement was categorized when more than 2 separate wounds in different limbs were operated upon or when debridement was extended to deep muscle layer or beyond. Minor wound debridement was when ≤2 separate wounds were debrided.

The following indices were used in the study:

- Cross match to transfusion ratio (C/T ratio) = number of units cross matched/number of units transfused. A ratio of 2.5 and below was considered indicative of significant blood usage.
- Transfusion probability (%T) = number of patients transfused/number of patients cross matched × 100%. A value of 30% and above was considered indicative of significant blood usage.
- Transfusion index (TI) = number of units transfused/number of patients cross matched. A value of 0.5 or more was considered indicative of significant blood utilization.

MSBOS was proposed based upon these calculations using Mead’s criteria [4, 14].

Data was analyzed using SPSS version 20.0 (Chicago, Illinois, USA). Qualitative variables were expressed as percentages while quantitative variables were represented as mean ± standard deviation. T-tests and Chi-square test were used where appropriate and *P*-value of < 0.05 was considered significant.

## Results

Six hundred and ninety-nine patients were included in the study population and their demographic characteristics are shown in Table 1. The mean age was found to be 50 ± 18 years. 60% of the study population were males and majority of the patients were ASA level II (54%). There were more elective as compared to emergency cases and tourniquet was used in majority of the procedures.

**Table 1:** Demographic characteristics of study population (*n* = 699)

Characteristics	No. of patients (%)/Mean ± S.D
Age (years)	50.23 ± 18.18
Gender	
Male	416 (59.5)
Female	283 (40.5)
ASA Status	
ASA I	126 (18.0)
ASA II	380 (54.4)
ASA III	166 (23.7)
ASA IV	25 (3.6)
Tourniquet Status	
Applied	410 (58.7)
Not applied	289 (41.3)
Nature of Surgery	
Emergency	286 (40.9)
Elective	413 (59.1)
Pre-operative Hb (g/dl)	12.17 ± 1.95

Overall, the calculated CT ratio was 4.87, transfusion index was 0.55 and transfusion probability was 25%. Table 2 shows the calculated CT ratios, transfusion index and transfusion probabilities for various orthopedic procedures. The highest CT ratio and lowest transfusion probability was calculated for arthroscopic procedures followed by total knee replacement, while tumor resection surgeries had the lowest CT ratio. On the basis of transfusion probabilities, the calculated MSBOS for each procedure is also given in Table 2.

**Table 2:** Outcome variables for various orthopaedic procedures

Procedure	Total Cases	Units of PCV^a^ Cross-matched	Units of PCV^a^ Transfused	CT^b^ Ratio	Transfusion Index	Transfusion Probability (%)	Calculated MSBOS
Total Hip Replacement	72	194	38	5.10	0.54	24.29	0.81
Total Knee Replacement	128	289	22	13.13	0.18	12.80	0.27
Open Reduction Internal Fixation	120	267	46	5.80	0.44	16.19	0.66
Closed Reduction Internal Fixation	145	351	42	8.36	0.31	25.0	0.46
Minor Wound Debridement	81	157	39	4.03	0.64	36.07	0.96
Major Wound Debridement	58	178	92	1.93	1.64	60.71	2.46
Tumor Resection	31	142	61	2.33	2.03	40.00	3.05
Arthroscopy	40	61	2	30.5	0.06	6.25	0.09
Hand and Microvascular Surgeries	24	60	7	7.5	0.29	25.00	0.44
Overall	699	1699	349	4.87	0.55	25.0	

^a^ *PCV*Packed Cell Volume

^b^*CT* Cross Match to Transfusion

The overall mean CT ratio was found to be significantly higher than the desired standard value of 2.5 (*P* = 0.036). However, the overall mean transfusion index and transfusion probability were found to be comparable to their standard values of 0.5 (*P* = 0.35) and 30% (*P* = 0.81) respectively. Total of 172 patients had a CT ratio of < 2.5; a compliance rate of 24.6% with the reference value.

A significant difference was noted when different procedures were compared with regards to their CT ratios being less or greater than 2.5 (*P* = < 0.001) as shown in Table 3. Major wound debridement had the highest proportion of patients (46%) with a CT ratio of < 2.5, whereas knee and hip replacement surgeries had a significantly lower proportion of patients in that group with 8.6 and 19.4% respectively. Amongst all cases of total hip replacements, 23.6% were done in an emergency setting while 76.4% were elective and no significant difference in CT ratio was found between them (*P*-value = 0.830). Mean age for patients with CT ratio < 2.5 was found to be significantly lower than for CT ratio > 2.5. In terms of gender, males were more likely to have a CT ratio of < 2.5 with the difference being statistically significant (*P* = < 0.001). Use of tourniquet was also found to have a significant association with CT ratio, while ASA status and surgery type showed no significant association (Table 3).

**Table 3:** Association of procedure and patient characteristics with CT ratio

Characteristics	CT Ratio (%)		*P*-value	Odds Ratio (95% CI)
	< 2.5	> 2.5		
Age (years)	46.02 ± 17.84	51.60 ± 18.10	< 0.001	
Procedure			< 0.001	
Total Hip Replacement	14 (19.4)	58 (80.6)		
Total Knee Replacement	11 (8.6)	117 (91.4)		
Open Reduction Internal Fixation	30 (25.0)	90 (75.0)		
Closed Reduction Internal Fixation	20 (13.8)	125 (86.2)		
Minor Wound Debridement	37 (45.7)	44 (54.3)		
Major Wound Debridement	36 (62.1)	22 (37.9)		
Tumor Resection	9 (29.0)	22 (71.0)		
Arthroscopy	9 (22.5)	31 (77.5)		
Hand and Microvascular Surgeries	6 (25.0)	18 (75.0)		
Gender			< 0.001	1.93 (1.33–2.80)
Male	122 (29.3)	294 (70.7)		
Female	50 (17.7)	233 (82.3)		
ASA Status			0.168	
ASA I	28 (22.2)	98 (77.8)		
ASA II	87 (22.9)	293 (77.1)		
ASA III	46 (27.7)	120 (72.3)		
ASA IV	10 (40.0)	15 (60.0)		
Surgery Type			0.255	
Emergency	64 (22.4)	222 (77.6)		
lective	108 (26.2)	305 (73.8)		
Use of Tourniquet			0.003	0.59 (0.42–0.83)
Yes	84 (20.5)	326 (79.5)		
No	88 (30.4)	201 (69.6)		

The variables were then analyzed using binary logistic regression model to control for co-variates and the target CT ratio of < 2.5 was coded as 1. After controlling for other variables, age, procedure performed, ASA status and use of tourniquet were found to be significantly associated with CT ratio as shown in Table 4. It was shown that ASA II patients were more likely to achieve a CT ratio of < 2.5 compared to ASA I; whereas ASA III and IV patients had no significant difference compared to ASA I.

**Table 4:** Logistic regression model for effect of baseline characteristics on CT ratio

Characteristics	*P*-value	95% Confidence Interval
Age	0.03	1.001–1.03
Procedure	< 0.001	0.77–0.93
Gender	0.13	0.91–2.11
ASA Status (reference ASA I)	0.01	
ASA II	0.007	1.47–10.96
ASA III	0.07	0.94–5.64
ASA IV	0.35	0.63–3.82
Surgery type	0.13	0.87–2.88
Use of torniquet	0.007	0.26–0.81

Current mean cost of blood cross match for each procedure was compared with mean cost according to MSBOS calculated, as shown in Table 5. The cost of 1 unit of PCV cross match at our institution was USD 48.4. Considerable reduction in cost was noted for each procedure with the highest difference shown for hand and microvascular surgeries, estimated at almost USD 100 per patient.

**Table 5:** Comparison of mean cost difference with implementation of MSBOS

Procedure	Total Cases	Mean units of PCV cross-matched	Mean Cost (USD^a^)	Mean Cost with MSBOS (USD^a^)	Cost Difference (USD^a^)
Total Hip Replacement	72	2.69	130.2	39.2	91
Total Knee Replacement	128	2.26	109.4	13.1	96.3
Open Reduction Internal Fixation	120	2.23	107.9	31.9	76
Closed Reduction Internal Fixation	145	2.42	117.1	22.3	94.8
Minor Wound Debridement	81	1.94	93.9	46.5	47.4
Major Wound Debridement	58	3.07	148.6	119.1	29.5
Tumor Resection	31	4.58	221.7	147.6	74.1
Arthroscopy	40	1.53	74.1	4.36	69.74
Hand and Microvascular Surgeries	24	2.5	121.0	21.3	99.7

^a^*USD* United States Dollar

## Discussion

Blood is among the top 10 most expensive liquids in the world. Despite a large amount of blood being donated free, it is the processing of blood after donation and the amount of work that goes into the process that makes it so expensive, thereby warranting effective utilization of this precious lifesaving liquid [3].

It is essential to manage blood bank resources and organize blood ordering practices in a way that results in effective utilization and minimal wastage of blood products. Hence, MSBOS is implemented universally to streamline ordering of blood for various surgeries and prevent wastage of resources including blood products, time and finances. It has been in use since 1975 and has undergone regular modifications as needed [7, 13].

In view of the results obtained, Table 6 shows our recommended MSBOS for the aforementioned procedures. Previously in 2001, Chawla et al. had established MSBOS for various surgical procedures at our institution. Multiple surgical specialties were accounted for in the study including general surgery, cardiothoracic, urology, otolaryngology as well as orthopedics. Amongst orthopedic procedures, they had proposed MSBOS of 1 unit, group and screen (G & S) and 2 units for total hip replacement, total knee replacement and open reduction internal fixation (ORIF) respectively [5]. In comparison, 14 years later, we also recommend similar MSBOS except for ORIF where our study suggests a lower MSBOS of 1 unit. This decrease can be attributed to advances in surgical techniques and hence reduced blood loss.

**Table 6:** Recommended MSBOS

Procedure	Recommended MSBOS
Total Hip Replacement	1
Total Knee Replacement	G & S^a^
Open Reduction Internal Fixation	1
Closed Reduction Internal Fixation	G & S
Minor Wound Debridement	1
Major Wound Debridement	2
Tumor Resection	3
Arthroscopy	G & S^a^
Hand and Microvascular Surgeries	G & S

^a^ *G & S* Group and Screen

Prior studies have constantly shown high rates of blood cross matching and underutilization of resources. A study held in Egypt in 2011 showed a similar overall CT ratio of 3.9 for a wide range of surgical procedures with individual CT ratios of as high as 10.6 for Urology – Endoscopy department [8]. Furthermore, international literature shows huge variations in CT ratios, ranging from < 0.5 to > 15 for some surgical procedures [2, 12, 17, 20].

The current results revealed total hip and knee replacements, arthroscopic surgeries and ORIF to have the worst blood transfusion parameters and therefore contributing highly to the wastage of blood products. Similarly, in a study from Thailand, Wong-Aek et al. also obtained CT ratios of 5.9 and 36 for total hip and knee arthroplasties respectively, considerably higher than our results. Subsequently, they recommended MSBOS of G & S for pre-operative haematocrit levels of above 33% and 36% for total knee and hip arthroplasties respectively. However, they reported no transfusions for arthroscopic procedures and therefore did not recommend G & S for arthroscopies [18]. In a study at John Hopkins Medical Institutions, total knee replacement had the 3rd highest CT ratio amongst orthopedic procedures below open shoulder and foot bone surgeries [6]. Subramanian et al. in 2010 reviewed blood ordering practices for orthopedic surgeries at a center in India and recommended similar MSBOS for hip replacement and internal fracture fixation surgeries [13]. In another study, Kumari et al. recommended MSBOS of 2 units each for total hip and bilateral knee replacements and G & S for unilateral knee replacement surgeries [9].

We also found significant associations between CT ratio, ASA status and use of tourniquet after controlling for other co-variables. ASA II patients were more likely to have a CT ratio of < 2.5 representing more blood usage in these patients compared to ASA I. However, considerable differences in percentage of patients with ASA I, II, III and IV may have led to biased results. Using tourniquet significantly decreased the likelihood of CT ratio being < 2.5. This relationship can be attributed to decreased blood loss with usage of tourniquet and hence decreased requirement of blood transfusion. Although there is debate in literature regarding effectiveness of tourniquet in reducing blood loss, the findings of this study favor its use for the same [15, 16]. A significant lower mean age was calculated for patients with CT ratio < 2.5, indicating a lower blood ordering practice for younger patients.

With regards to financial benefits, a considerable reduction in cost was noted per patient if calculated MSBOS were to be followed. This decrease is of utmost importance for a developing country like Pakistan, where majority of the patients belong to lower socio-economic status and health expenditure is entirely out of pocket. In addition, this decrease in cost by virtue of compliance to MSBOS, can also lead to better access to care as a result of increased availability of finances.

The CT ratio of the current study is significantly higher than the recommended value, indicating significant over ordering of blood products pre-operatively. However, transfusion probability was comparable to the standard recommendation representing that a significant number of patients ended up requiring blood transfusions. There was also adequate blood usage with transfusion index being as high as 0.55 [12]. Previous studies have also shown similar results with high CT ratios but comparable transfusion index or probabilities to the recommendations [8, 12, 17].

Our survey reveals severe lack of compliance to previously recommended MSBOS at our institution. Despite setting up recommendations for multiple procedures in 2001, the CT ratios were well above standards in the department of orthopedics. Internationally, compliance with recommended MSBOS has shown to decrease CT ratios significantly and result in effective utilization of blood products [11, 18, 19]. Hence, routine yearly auditing of blood ordering practices in orthopedics as well as other departments is warranted at our institution to ensure compliance with the recommendations. In addition, there is also a need for separate studies for each specialty to update the MSBOS proposed in 2001 and to add procedures not included previously. Furthermore, whether the utilized blood was really indicated or not is another area for research. In a prospective study conducted at our institute to determine the proportion of inappropriate transfusions amongst patients undergoing orthopedic surgery, it was revealed that within the 126 included patients, 33% were transfused appropriately while 65% were inappropriate transfusions [1].

To the best of our knowledge, this is the first study in Pakistan encompassing all major orthopedic procedures with a large enough sample size to calculate MSBOS. Moreover, the results obtained substantiate the findings of a prior study at the same institute and are also comparable to results from similar parts of the world i.e. India and Thailand. However, the retrospective nature of the study resulted in only accounting for the blood products being cross matched by the blood bank when calculating ordered blood. It often occurs that the blood bank is unable to process all our requests due to unavailability of blood, and therefore, the results can be more dismal if actual units of PCV ordered were to be accounted for. Other limitation of this study is the classification of orthopedic procedures which is over simplified. For example, in the category of ORIF, we could not go through specific procedures and whether the fracture was open, complicated or with soft tissue damage. Similarly, for CRIF, the specific type of fracture and use of implant is not mentioned.

Hence, there is still need for more large scale and prospective studies to establish MSBOS and ensure its compliance. Nevertheless, we would recommend the use of currently proposed MSBOS across the country until other centres conduct similar trials and establish their own criteria.

## Conclusion

Results of this study show significant wastage of blood products and non-compliance with blood ordering guidelines. Measures need to be taken for standardization and implementation of blood ordering practices hence conserving this important commodity.

## Abbreviations

%T: Transfusion probability
AKUH: Aga Khan University hospital
ASA: American society of anesthesiologists
CT ration: Cross match to transfusion ratio
G & S: Group and screen
Hb: Hemoglobin
MSBOS: Maximum surgical blood order schedule
ORIF: Open reduction internal fixation
PCV: Packed cell volume
TI: Transfusion index
USD: United States Dollar
WME: Wide margin excision
